# Loss of mitochondrial ClpP, Lonp1, and Tfam triggers transcriptional induction of *Rnf213*, a susceptibility factor for moyamoya disease

**DOI:** 10.1007/s10048-020-00609-2

**Published:** 2020-04-28

**Authors:** Jana Key, Antonia Maletzko, Aneesha Kohli, Suzana Gispert, Sylvia Torres-Odio, Ilka Wittig, Juliana Heidler, Clea Bárcena, Carlos López-Otín, Yuanjiu Lei, A. Phillip West, Christian Münch, Georg Auburger

**Affiliations:** 1 Experimental Neurology, Goethe University Medical School, 60590 Frankfurt am Main, Germany; 2 Faculty of Biosciences, Goethe-University, Frankfurt am Main, Germany; 3 Functional Proteomics Group, Goethe-University Hospital, 60590 Frankfurt am Main, Germany; 4 Departamento de Bioquímica y Biología Molecular, Facultad de Medicina, Instituto Universitario de Oncología, Universidad de Oviedo, 33006 Oviedo, Spain; 5 Department of Microbial Pathogenesis and Immunology, Texas A&M University, College Station, TX USA; 6 Institute of Biochemistry II, Goethe University Medical School, 60590 Frankfurt am Main, Germany

**Keywords:** Perrault syndrome, Mitochondrial dysfunction, AAA+ disaggregase, Ubiquitin ligase, Stroke genetics, Innate immunity, Autoimmune vasculopathy

## Abstract

Human *RNF213*, which encodes the protein mysterin, is a known susceptibility gene for moyamoya disease (MMD), a cerebrovascular condition with occlusive lesions and compensatory angiogenesis. Mysterin mutations, together with exposure to environmental trigger factors, lead to an elevated stroke risk since childhood. Mysterin is induced during cell stress, to function as cytosolic AAA+ ATPase and ubiquitylation enzyme. Little knowledge exists, in which context mysterin is needed. Here, we found that genetic ablation of several mitochondrial matrix factors, such as the peptidase *ClpP*, the transcription factor *Tfam*, as well as the peptidase and AAA+ ATPase *Lonp1*, potently induces *Rnf213* transcript expression in various organs, in parallel with other components of the innate immune system. Mostly in mouse fibroblasts and human endothelial cells, the *Rnf213* levels showed prominent upregulation upon Poly(I:C)-triggered TLR3-mediated responses to dsRNA toxicity, as well as upon interferon gamma treatment. Only partial suppression of *Rnf213* induction was achieved by C16 as an antagonist of PKR (dsRNA-dependent protein kinase). Since dysfunctional mitochondria were recently reported to release immune-stimulatory dsRNA into the cytosol, our results suggest that mysterin becomes relevant when mitochondrial dysfunction or infections have triggered RNA-dependent inflammation. Thus, MMD has similarities with vasculopathies that involve altered nucleotide processing, such as Aicardi-Goutières syndrome or systemic lupus erythematosus. Furthermore, in MMD, the low penetrance of *RNF213* mutations might be modified by dysfunctions in mitochondria or the TLR3 pathway.

**Electronic supplementary material:**

The online version of this article (10.1007/s10048-020-00609-2) contains supplementary material, which is available to authorized users.

## Introduction

The correct folding of proteins is crucial for their function and their potential toxicity, so cells have developed several sophisticated pathways dedicated to “unfolded protein response” (UPR). The UPR as a quality-control system was described for several subcellular compartments, such as the cytosol, the endoplasmic reticulum (ER), and most recently mitochondria (mtUPR) [1–3]. The mtUPR was first described in *C. elegans* and is being intensely investigated in mammals [4]. Experiments in *C. elegans* demonstrated that a key role in mtUPR is played by the mitochondrial matrix peptidase ClpP (caseinolytic peptidase P), which has a conserved function since *E. coli* bacteria until mammalian mitochondria to degrade peptides with improper folding as they emerge from ribosomal translation or the mitochondrial import pore [5–9].

Loss-of-function mutations in the human *ClpP* gene, which is mainly responsible for mitoribosome folding quality [10], lead to an autosomal recessively inherited disease called Perrault syndrome type 3 (PRLTS3) [11]. The deficiency of ClpP in mice results in the accumulation of the AAA+ ATPase ClpX, probably as direct protein interaction effect, as well as accumulation of mitochondrial DNA and RNA [12–14] that are known triggers of innate immunity [15, 16]. Probably as a consequence, a signature of many inflammatory factors and several subunits of the immunoproteasome was upregulated in oligonucleotide microarray transcriptome profiles of *ClpP*^−/−^ heart, liver, and brain [12]. Within this inflammatory signature, an expression induction was observed for the cytosolic (also nuclear) AAA+ ATPase named mysterin (encoded by *Rnf213*). This completely novel insight was intriguing, while also raising questions: firstly, here a mitochondrial dysfunction, which will normally trigger respiratory failure together with a deficient breakdown of glucose, amino acids, and fatty acids, acts to activate the expression of Mysterin as a known coordinator of angiogenesis [17–19], influencing how tissues are supplied by nutrients, oxygen, and immune cells. Secondly, Mysterin contains domains for protein disaggregation/degradation, as well as homo-oligomerization features to form rings, so it could play a compensatory role in UPR, although it remains unclear how its localization in the cytosol [20] would aid the unfolded protein response within *ClpP*^−/−^ mitochondria [17, 18, 21, 22].

Since mitochondria descend from endosymbiotic bacteria, their DNA and protein have bacterial features, such as hypomethylation of nucleotides as well as formyl-methionine at the N-terminus of peptides. If either of them is released into the cytosol, the innate immune defense of any cell will be activated via its diverse pattern-recognition-receptors such as Toll-Like-Receptors (TLR1–11). It is unknown, which specific properties of the mtUPR make compensation by the cytosolic ubiquitin-proteasome/autophago-lysosome degradation pathways ineffective, so that mysterin and the immunoproteasome are being induced.

There are several studies implicating mysterin in immunity [21, 23, 24]. Mysterin is also linked to inflammatory pathways that are connected to hypoxia-related vascular changes [17, 21, 25]. The hypoxia-inducible transcription factor-1 (HIF-1) is the main transcription factor that gets activated by low oxygen levels. This activation is tightly linked to the NFkB pathway [26] and also induced via the double-stranded RNA–dependent protein kinase (PKR, also known as EIF2AK2) pathway [27]. PKR was described to be involved in inflammatory events within cells and to depend on interferon gamma (IFNG), displaying one of the first lines of defense against RNA-viruses in the innate immune pathways [28, 29]. Overall, the genetic interaction of ClpP with mysterin appeared plausible.

We now focused on this consistent and strong transcriptional activation of *Rnf213* in more detail. *Rnf213* is the main susceptibility gene for moyamoya disease (MMD) [22, 30], a specific intracranial vascular disorder characterized by progressive, occlusive lesions of internal carotid arteries and branches in the circle of Willis, resembling a puff of smoke (upon contrast angiography) that is called “moyamoya” in Japanese [31–33]. MMD is currently recognized as one of the major causes of stroke in children [34]. It is not clear if the lesions of blood vessels and brain are due to affection of endothelial, smooth muscle, adventitia fibroblast, barrier glia, or neural cells.

The gene *Rnf213* encodes for a mainly cytosolic protein with a RING finger motif and AAA+ ATPase domain, so the RNF213 protein was also called mysterin (moyamoya steno-occlusive disease-associated AAA+ and RING finger protein) [35]. *Rnf213* is conserved across vertebrates, with ubiquitous expression in human and murine tissues [22, 30]. The structure of RNF213 with its two AAA+ ATPase modules is similar to bacterial ClpB [35]. In mammals, ClpB is a mitochondrial molecular chaperone that cooperates with HSP70 in the physical disaggregation of protein aggregates, thus contributing to cellular proteostasis [35]. However, it remains unclear whether RNF213 also has disaggregase functions, its physiological roles and protein substrates remain elusive.

To corroborate the role of ClpP specifically and of mitochondrial dysfunction in general regarding mysterin expression regulation, it was tested if *Rnf213* induction occurs also in response to other mitochondrial mutations. We analyzed fibroblasts with mutation of the other mitochondrial matrix protease, Lonp1, which is mainly responsible for respiratory chain assembly [36, 37], and fibroblasts with mutation of the mitochondrial transcription factor A (TFAM), which is responsible for mitochondrial RNA biogenesis [38, 39]. To elucidate which aspects of mitochondrial dysfunction trigger *Rnf213* transcriptional induction, we tested four alternative hypotheses in mouse embryonal fibroblasts (MEF), human umbilical vein endothelial cells (HUVEC), or human neuroblastoma cells (SH-SY5Y):

1. Dysfunctional mitochondria become cytotoxic via mitochondrial precursor overaccumulation stress (mPOS) [40]. This was modeled by administration of the uncoupling agent FCCP, which impairs mitochondrial import so that proteins destined for the mitochondrial matrix accumulate in the cytosol, triggering cell death.
2. Dysfunctional mitochondria limit cell viability when their production of nutrients, iron-sulfur-clusters, and heme is insufficient [41]. This nutrient deprivation aspect and the relevance of macroautophagy pathways were analyzed by investigating the effect of a starvation protocol on cellular *Rnf213* expression.
3. Dysfunctional mitochondria may become similarly toxic as invading bacteria, activating the innate immune defenses against prokaryotic formyl-peptides and other damage-associated molecular patterns [42, 43]. *Rnf213* is known to be involved in innate immunity processes as a ubiquitin ligase, possibly responsible for the turnover of inflammatory factors. Thus, its regulation was assessed after the administration of the bacterial cell wall component LPS (lipopolysaccharides), Pam3CSK4, and IFNG.
4. Dysfunctional mitochondria release mtDNA and dsRNA from their matrix into the eukaryotic cytosol [13, 14]. *ClpP*^−/−^ cells show an excessive accumulation of mitochondrial DNA and RNA as previously observed [12], and this might trigger an unusually strong immune activation. For a selective analysis of the responsiveness of the innate immune pathways towards hypo-methylated nucleotides, the toxic DNA-analogue CpG and the toxic RNA-analogue Poly(I:C) were employed.

Thus, our study focused on the novel observation that mitochondrial mutations modulate the nuclear transcription of *Rnf213*; it aimed to elucidate the trigger factors and signaling mechanisms involved, as well as the relevance for several cerebrovascular cell types. The results suggest that RNA toxicity more than protein toxicity is relevant for MMD pathogenesis that may originate from infectious pathogens or from cellular dysfunctions.

## Results

### Rnf213 is consistently upregulated in the global transcriptome and proteome of ClpP^−/−^ mice

We wanted to understand how the loss of ClpP causes molecular changes and to identify the relevant compensatory efforts among them. Therefore, previously published global transcriptome profiles of *ClpP*^−/−^ mouse brain, heart, and liver at the age of 9–10 months were now analyzed further (compare Table S2 of Gispert et al., 2013 [12]).

**Fig. 1:**
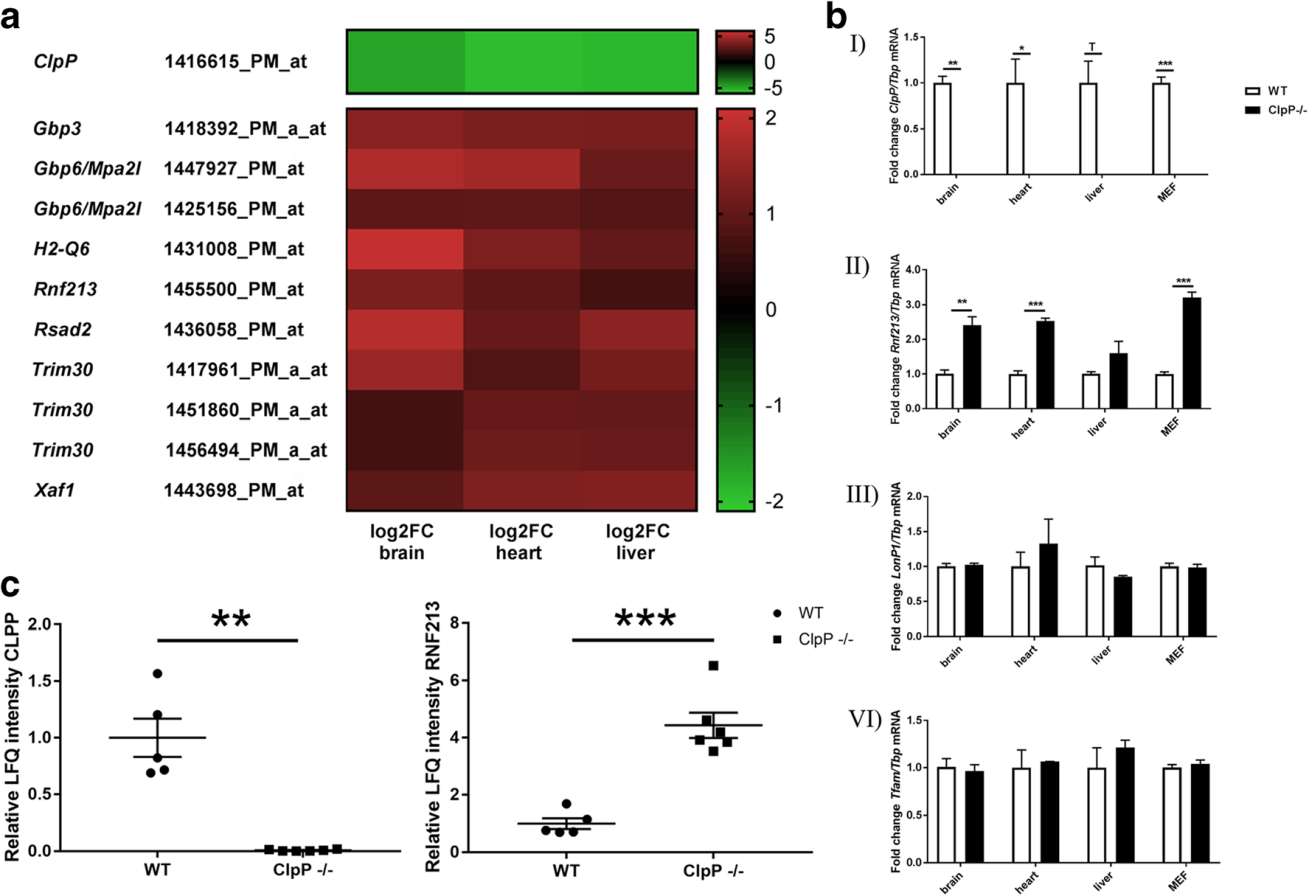
Expression changes of *Rnf213* in ClpP-deficient tissue. **a** Heatmap, extracting the changed transcript levels for *ClpP* and selected anti-infection defense factors from a global Affymetrix microarray screen, that was published before [12]. Significant dysregulations are highlighted with different red color, if their logarithmic fold-change (log_2_FC) exceeds 0.6 as cutoff; the panel shows gene symbols, the Affymetrix oligonucleotide number under study, and the color-graded expression changes, illustrating upregulations in red, downregulations in green. Among other immunological factors, *Rnf213* appeared upregulated at the transcript level in brain, heart, and liver tissue. **b** RT-qPCR results showing mRNA abundances relative to *Tbp* transcript levels in ClpP-deficient mouse brain, heart, liver tissue, and MEF. *n* = 3–4 for each, WT and *ClpP*^−/−^, showing biological replicates. Transcript levels are documented for (I) *ClpP*, confirming the genetic ablation in all tissues; (II) *Rnf213*, showing upregulation in brain, heart, and MEF; (III) *Lonp1*, which is transcriptionally not affected by the loss of *ClpP*; and (IV) the mitochondrial transcription factor *Tfam,* also not changed in the mutant tissues. **c** Protein abundance of CLPP (left panel) and RNF213 (right panel) in brain proteome of 5 WT versus 6 *ClpP*^−/−^ mice. LFQ, label-free quantification value

In the global transcriptome of mice with deficiency of the mitochondrial matrix peptidase ClpP, upregulations of alternative protein degradation factors would be expected. The only one such upregulated factor observed, mysterin, contains an AAA+ ATPase domain and also an E3 ubiquitin ligase motif. Figure 1 a provides a synopsis of *Rnf213* mRNA dysregulation with fold-changes (FC), among other innate immunity factors in three *ClpP*^−/−^ tissues. *Rnf213* appears upregulated in brain hemisphere (log_2_FC = 1.25, FC = 2.38), liver (log_2_FC = 0.71, FC = 1.64), and heart (log_2_FC = 0.96, FC = 1.95) tissues.

Validation experiments in independent animals and MEF by reverse-transcriptase real-time quantitative polymerase chain reaction (RT-qPCR) were conducted (fold-changes and *p-* values for all expression analyses in the diverse figures are summarized in Suppl. Table S1). In Fig. 1b (panel I), the genetic ablation of *ClpP* was confirmed in brain, heart, liver, and MEF. Panel II verified significant *Rnf213* mRNA upregulations (3 wild type versus 3 mutants at the age of 7–10 months) in brain hemisphere (2.41-fold; *p* = 0.0178), heart (2.33-fold; *p* = 0.0132), and MEF (3.20-fold; *p* = 0.0003). In liver tissue *Rnf213* was not significantly changed (1.60-fold; *p* = 0.2250), possibly reflecting tissue specificity.

The accumulation of non-degraded proteins in the mitochondrial matrix might be compensated by the upregulation of Lonp1, which is the other mitochondrial matrix peptidase involved in protein quality control, respiratory-complex assembly, gene expression, and mitochondrial stress response [36]. However, RT-qPCR showed that the *Lonp1* transcript levels were not significantly altered in the absence of ClpP (Fig. 1b, panel III). Similarly, the accumulation of mtDNA and mtRNA in ClpP-deficient tissues might lead to a dysregulation of the mitochondrial transcription factor A (TFAM). It was recently observed [42] that the heterozygous loss of *Tfam* activates innate immunity pathways. In a published transcriptome analysis of *Tfam*^+/−^ MEF, *Rnf213* was reported to appear with other inflammatory factors among the upregulated transcripts [42]. In order to exclude that the transcriptional induction of *Rnf213* in ClpP mutant tissues is indirectly due to *Tfam* expression changes in response to excess mtDNA, *Tfam* transcript levels were assessed in *ClpP*^−/−^ tissues by RT-qPCR. *Tfam* mRNA was unchanged in brain, liver and heart tissues or MEF in the absence of ClpP (Fig. 1b, panel IV). Next, we asked if the transcriptional activation of *Rnf213* also translates to higher RNF213 protein levels. Indeed, they were elevated 4.4-fold (*p* = 0.0002) in the *ClpP*^−/−^ brain global proteome, as demonstrated by label-free quantitative mass spectrometry in the brain of *ClpP*^−/−^ mice (5 wild type vs. 6 mutants, Fig. 1c).

Altogether, it was documented that Rnf213 is upregulated between 2.3- and 4.4-fold at both, protein and transcript level, in a mouse mutant where the mitochondrial matrix peptidase *ClpP* is absent. This induction contrasts with the unchanged levels of *Lonp1* and *Tfam*.

### Rnf213 expression dysregulation in the presence of various stressors, including mitochondrial uncoupling, starvation, Poly(I:C), LPS, and IFNG

The protein RNF213 is associated with pathways that react upon different types of stress, such as inflammation and hypoxia [17, 21, 25]. Thus, different stress conditions were applied to murine bone-marrow-derived macrophages (BMDM), murine embryonal fibroblasts (MEF), the human neuroblastoma cell line (SH-SY5Y), and human umbilical vein endothelial cells (HUVEC), documenting the subsequent regulation of *Rnf213* mRNA.

For an initial survey, BMDM as the principal tool of immunological research were exposed to compounds that are known to trigger separate toll-like receptor (TLR) pathways such as the TLR4 agonist LPS, the TLR1/TLR2 agonist Pam3CSK4 and the TLR9 agonist CpG. A massive transcriptional response of *Rnf213* (4.98-fold, *p* < 0.0001) together with *Rsad2* and *Ddx58* mRNAs as innate immunity factors was observed after LPS administration over 6 h in vitro, a response that was significantly enhanced by the absence of ClpP (9.00-fold, *p* < 0.0001). Conversely, *ClpP* mRNA levels decreased to 31% in WT BMDM in reaction to LPS treatment (*p* < 0.0001) (Fig. S1).

We switched to relatively stress-resistant primary fibroblasts, to stress-susceptible neural cells and endothelial cells, in an effort to understand the brain and blood vessel affection in vitro. First, the mitochondrial uncoupling agent FCCP was used as a stressor. However, neither murine *Rnf213* was changed in MEF after 24 h (Fig. S2a), nor human *RNF213* in neuroblastoma SH-SY5Y cells after 24 h and 36 h (Fig. S2b). In endothelial HUVEC cells, *RNF213* transcript was induced 2.13-fold (*p* = 0.0072). Thus, FCCP-triggered reduction of the mitochondrial membrane gradient DeltaPsi(m), which would lead to mPOS and protein aggregation outside the mitochondrial import pores, is not sufficient for *Rnf213* upregulation in murine fibroblasts and human neuronal cells, whereas endothelial cells appear exceptionally responsive to this alteration of mitochondrial homeostasis.

**Fig. 2:**
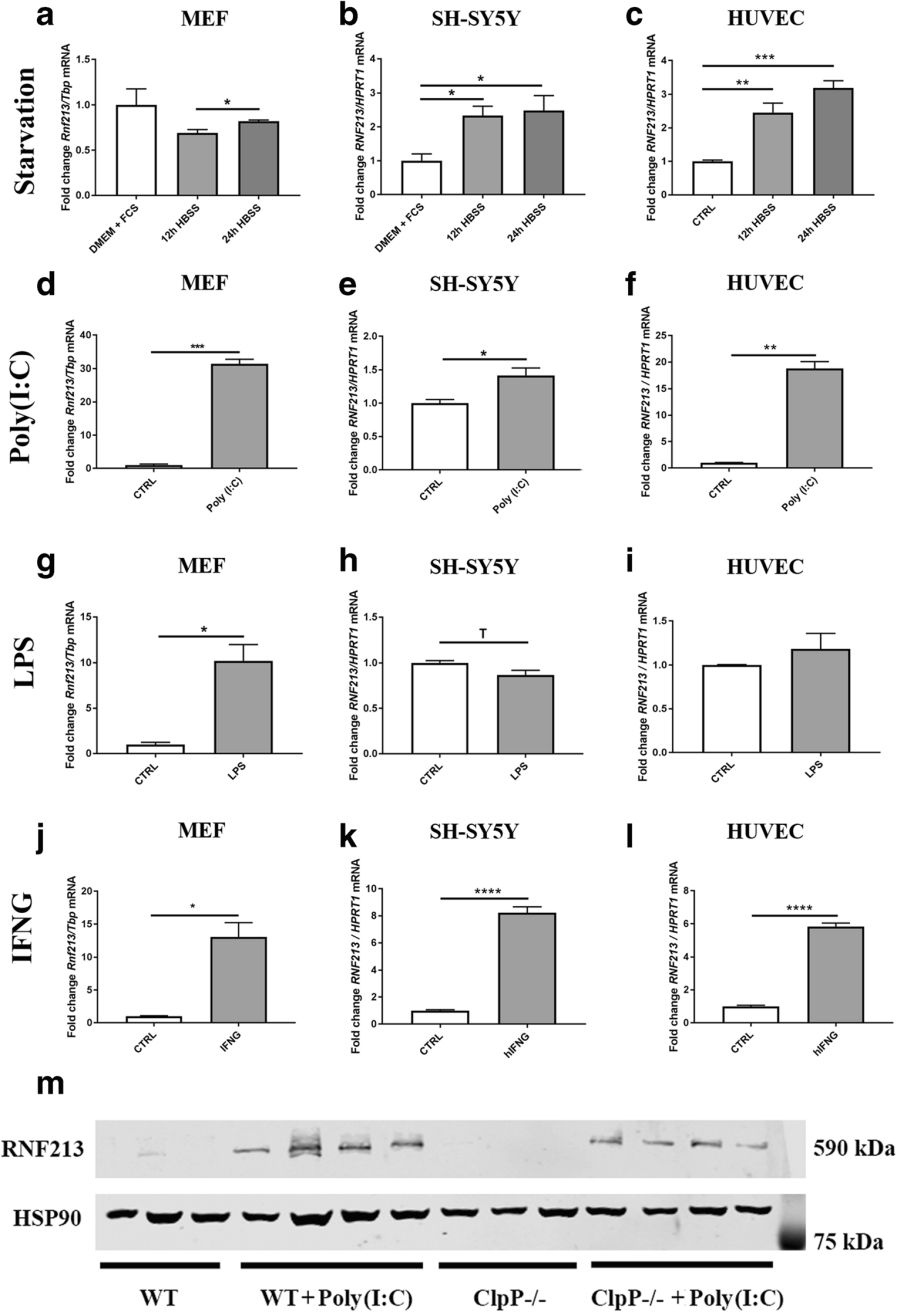
RT-qPCR analyses of wild-type MEF, human neuroblastoma SH-SY5Y cells, and human umbilical vein endothelial cells (HUVEC) for the expression of *Rnf213* after exposure to different stress situations. *Rnf213* transcript in **a** MEF, **b** SH-SY5Y, and **c** HUVEC cells after serum starvation (DMEM, Dulbecco’s modified Eagle medium; FCS, fetal calf serum; HBSS, Hank’s balanced salt solution; CTRL, untreated control) for indicated times. *Rnf213* transcript in **d** MEF, **e** SH-SY5Y, and **f** HUVEC cells is quantified after application of the pathogenic dsRNA analog Poly(I:C) for 16. *Rnf213* transcript in **g** MEF, **h** SH-SY5Y, and **i** HUVEC cells is quantified after incubation with the bacterial cell wall component Lipopolysaccharide (LPS) for 24 h. R*nf213* transcript in **j** MEF, **k** SH-SY5Y, and **l** HUVEC cells is quantified after incubation with murine or human interferon gamma (IFNG). The *Y*-axis of each plot shows the ratio of a transcript of interest versus mouse *Tbp* or human *HPRT1* as loading control. The bar graphs show mean and standard error of the mean (SEM), illustrating the significances with asterisks (Trend T 0.05 < *p* < 0.1; *p < 0.05, **p < 0.01, *** *p* < 0.001, **** *p* < 0.0001). **m** Quantitative immunoblot for RNF213 protein expression in untreated WT and *ClpP*^−/−^ MEF cells, and after incubation with Poly(I:C) at 1 μg/ml for 16 h. HSP90 served as loading control

The starvation of MEF over 24 h (Fig. 2a) in HBSS medium (which is devoid of amino acids and has only low glucose levels) without FCS (absence of trophic factors and lipids) resulted in a slight, yet not significant, reduction of *Rnf213* transcript by 0.69-fold compared to nutrient-abundant control conditions after 12 h (*p* = 0.1603). This was followed by a 0.82-fold change after 24 h (*p* = 0.3698), compared to control. The upregulation between 12 and 24 h was statistically significant (*p* = 0.0216). In contrast, upon exposure of neural SH-SY5Y cells to starvation conditions in HBSS medium, the expression of human *RNF213* was induced up to 2.33-fold at 12 h (*p* = 0.0260) and to 2.48-fold after 24 h (*p* = 0.0183) (Fig. 2b) compared to control conditions. Similarly, in HUVEC, *RNF213* was transcriptionally induced to 2.46-fold (*p* = 0.0024) after 12 h and up to 3.19-fold (*p* = 0.0001) after 24 h starvation (Fig. 2c). Thus, neural and endothelial cells appeared quite susceptible to nutrient deprivation, as opposed to fibroblasts that appear not vulnerable and less needful of cytosolic AAA+ disaggregase capacity when a period of nutrient shortage reduces protein synthesis. However, this steady increase over the whole observation period did not parallel the phasic transcriptional regulation of known mitophagy factors like *PINK1* and *PARK2*, which reaches a 3-fold maximum at 12–16 h and comes back to baseline levels at 48 h in SH-SY5Y cells [44]. Thus, it is doubtful whether *RNF213* is induced as a typical factor of the macroautophagy pathway.

In addition to a bacterial cell wall component like LPS, infection-like conditions in cell culture can also be mimicked by application of toxic RNA that simulates viral invasion. Thus, we exposed cells to a synthetic analog of dsRNA acting as TLR3 agonist, which is known as Poly(I:C), in comparison to LPS. In MEF the presence of Poly(I:C) for 16 h led to a massive 31-fold induction of *Rnf213* (*p* = 0.0012) (Fig. 2d). Less strongly, SH-SY5Y cells after 16 h of incubation with Poly(I:C) responded with an 8-fold induction of *Rnf213* (*p* = 0.0072) (Fig. 2e)*.* Again in a massive response as seen for MEF, the HUVEC cells induced *RNF213* mRNA 18.84-fold (*p* = 0.0047) (Fig. 2f).

Comparing viral pseudo-infection with bacterial pseudo-infection, the presence of LPS in MEF led to a 10-fold (*p* = 0.0330) induction of *Rnf213* (Fig. 2g). Interestingly, SH-SY5Y cells responded with a slight downregulation of *RNF213* in the presence of LPS (0.87-fold, *p* = 0.0549) (Fig. 2h). This lack of response can be explained by TLR4 absence in SH-SY5Y cells, see https://www.proteinatlas.org/. Also in HUVEC, the administration of LPS had much weaker effects on the transcriptional regulation of *RNF213* (Fig. 2i) than in the presence of abnormal RNA.

Following the track of infection-like state in cells, it is known that *Rnf213* is transcriptionally induced in endothelial cells via PKR (double-strand-RNA dependent protein kinase) after exposing the cells to IFNG [17]. IFNG is an important cytokine in the host defense against infection by viral and microbial pathogens. The promoter sequence of the human *RNF213* gene contains predicted binding sites for IRF1/2/4/9 and STAT1/2/3/5A (see *RNF213* entry in the GeneCards database), so RNF213 seems to be one among many interferon-stimulated genes. Upon treatment with IFNG, we observed *Rnf213* to be upregulated 13.05-fold (*p* = 0.0113) in MEF (Fig. 2j); furthermore, *RNF213* mRNA was also induced 8.23-fold (*p* < 0.0001) in SH-SY5Y cells (Fig. 2k), and 5.83-fold (*p* < 0.0001) in HUVEC (Fig. 2l). This confirms the already known dependence of *Rnf213* transcriptional response to IFNG in endothelial cells and reveals it to occur also in fibroblasts and neuronal cells.

These data indicate that the mysterin activation occurs prominently in endothelial cells and fibroblasts that are exposed to toxic RNA as it can be found during viral infections. This induction is stronger than during bacterial pseudo-infection stress or nutrient deprivation, which would all lead to unfolded protein responses.

### RNF213 protein is induced in MEF after exposure with Poly(I:C)

Thus, Poly(I:C) treatment results in transcriptional induction of *Rnf213*. This induction resulted in RNF213 protein becoming detectable by quantitative immunoblots both, in WT MEF and *ClpP*^−/−^ MEF (Fig. 2m). However, we were not able to demonstrate the stronger induction of RNF213 protein in the absence of ClpP. This could be attributed to a long delay in the synthesis of this large-size protein with 591 kDa, to technical difficulties (its large size makes the transfer to blotting membrane less linear) and possibly to turnover effects. To evaluate the turnover of RNF213 protein in the presence and absence of ClpP, we determined the protein abundance over a time course of 72 h by pSILAC with TMT-labeled mass spectrometry (Suppl. Fig. S3). After 72 h, RNF213 was degraded to 50%, but the decay rate of RNF213 did not differ between WT and *ClpP*^−/−^ MEF (Suppl. Fig. S3).

These results confirm that RNF213 plays an important role in the antiviral innate immune response and that Poly(I:C) may be the most appropriate stressor to induce mysterin at the mRNA and protein level, in comparison to LPS.

### Rnf213 induction via PKR pathway and its repression by the PKR inhibitor C16

There is a previous report that the signaling pathway downstream of protein kinase R (PKR, also known as EIF2AK2 or “interferon-induced, double-stranded RNA-activated protein kinase”) is involved in the transcriptional activation of *Rnf213* in endothelial cells [17]. This candidate pathway was assessed now, comparing murine embryonal fibroblasts and human neuroblastoma cells with HUVEC.

**Fig. 3:**
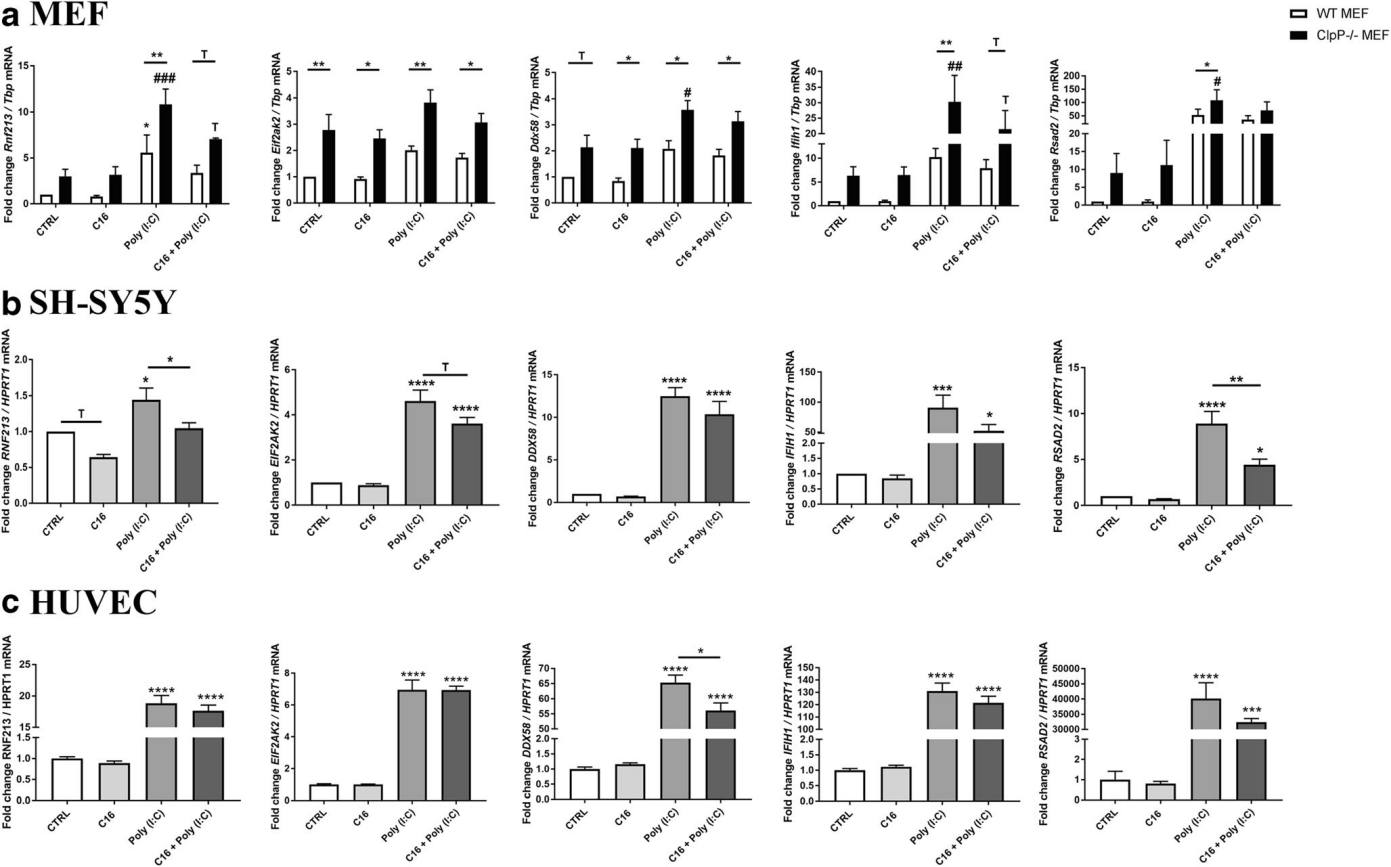
RT-qPCR analyses of innate immunity key factors in **a** MEF from WT (white bars) and *ClpP*^−/−^ (black bars) mice after incubation with the toxic RNA analog Poly(I:C), the PKR-inhibitor C16 and both substances in combination, **b** SH-SY5Y cells and **c** HUVEC cells. Transcript levels are normalized against endogenous murine *Tbp* or human *HPRT1* levels and are shown relative to untreated control conditions. Graphs show statistical results of 1-way ANOVA or 2-way ANOVA. (Trend T 0.05 < *p* < 0.1; * or #*p* < 0.05; ** or ##*p* < 0.01; *** or ###*p* < 0.001, ****p < 0.0001). Asterisks show significance compared to untreated WT, hashtags represent significance compared to untreated *ClpP*^−/−^ samples

Figure 3 a shows *Rnf213* expression in WT MEF compared to *ClpP*^−/−^ MEF after incubation either with Poly(I:C) alone, with the PKR inhibitor drug C16 or the combination of both substances. The shorter treatment period resulted in smaller transcriptional responses, saturation kinetics were avoided in this manner. *Rnf213* was induced 5.57-fold (*p* = 0.0307) in WT MEF upon the application of Poly(I:C) for 6 h. The *ClpP* null genotype triggered an even bigger induction of *Rnf213* in the presence of Poly(I:C) alone (10.84-fold; *p* = 0.0004) with a significant difference between WT and *ClpP*^−/−^ (*p* = 0.0099). In the combined presence of Poly(I:C) and C16, *Rnf213* was induced 3.38-fold (*p* = 0.3972) in WT and 7.04-fold (*p* = 0.0613) in *ClpP* null MEF, with a trend towards higher levels upon absence of ClpP (*p* = 0.0919).

Furthermore, the transcriptional changes of *PKR* (*Eif2ak2*) itself were tested, as well as of three cytosolic RNA/DNA sensors with antiviral functions (*Ddx58*, *Ifih1*, *Rsad2*). The results showed massive inductions of an antiviral state in the absence of *ClpP*^−/−^. This could already be seen in untreated conditions between wildtype and mutant cells, and all sensors were highly induced in the presence of Poly(I:C). In fibroblasts, these signaling pathways seemed to be modulated not only by PKR, since the presence of C16 did not significantly reduce the expression inductions of *Ddx58*, *Ifih1*, and *Rsad2*.

Because fibroblasts are relatively resistant to metabolic stress situations, we applied C16, Poly(I:C), and their combination to human neuronal SH-SY5Y cells and endothelial HUVEC cells. Figure 3 b shows that the addition of Poly(I:C) to the cell culture medium of SH-SY5Y cells for 10 h again induced *RNF213* transcripts in cells treated only with Poly(I:C) (1.44-fold; *p* = 0.0187). In cells with C16 alone, *RNF213* showed a trend towards reduction (0.64-fold, *p* = 0.0650). The combination of both substances resulted in the blunting of RNF213 induction with levels that were similar to control conditions (1.05-fold, *p* = 0.9841). In comparison to the *RNF213*-induced state with Poly(I:C) alone, this was a significant reduction (*p* = 0.0377). *PKR/ EIF2AK2* itself was not significantly changed at the mRNA level after the application of C16 (0.88-fold, *p* = 0.9903). Its induction by the combination of Poly(I:C) and C16 (3.62-fold, *p* < 0.0001) was less strong than with Poly(I:C) alone (4.61-fold, p < 0.0001), but both were highly induced. Again, the expression levels of the antiviral genes *DDX58*, *IFIH1* and *RSAD2* were tested. As expected, in SH-SY5Y cells Poly(I:C) led to the induction of an antiviral state, with DDX58 being induced 12.49-fold (*p* < 0.0001), IFIH1 90.55-fold (*p* < 0.0001), and RSAD2 8.90-fold (*p* < 0.0001). In SH-SY5Y, however, one factor with significant changes in the presence of the C16 was *RSAD2*, whose induction by Poly(I:C) was much weaker during PKR-inhibition (*p* = 0.0029). Thus, in neuronal cells, the PKR inhibition was relevant for *RNF213* and *RSAD2* expression regulation.

In Fig. 3c, the response of HUVEC cells to Poly(I:C) alone and in the presence of the PKR-inhibitor C16 is shown. *RNF213* got induced 18.84-fold (*p* < 0.0001) by Poly(I:C), but its expression was not reduced by the presence of C16. The same pattern was seen for *EIF2AK2*, *IFIH1*, and *RSAD2*. However, the expression of the cytosolic nucleic acid sensor *DDX58* was induced 65.34-fold (*p* < 0.0001) after incubation with Poly(I:C) and this very strong effect was significantly diminished in the presence of C16 (*p* = 0.0245).

These findings show that PKR pathway inhibition antagonizes the Poly(I:C) effect on the transcriptional regulation of *RNF213* in neural cells, but not in HUVEC and fibroblasts. Importantly, in fibroblasts, the RNA toxicity triggered by the addition of Poly(I:C) to the culture medium was further potentiated by ClpP deficiency with its known impairment of mitoribosome quality [10].

### MEF with diverse mitochondrial mutations show induction of Rnf213 and immunity factors

To corroborate that mitochondrial dysfunction in a more general sense and not only by the absence of ClpP influences *Rnf213* transcript regulation, and to test by a different approach if mitochondrial respiration or mitochondrial nucleotides are more important, MEF cells with mutations in additional mitochondrial factors were studied. In comparison to the mitochondrial matrix peptidase ClpP that is responsible for the turnover of mitoribosomes [10], we studied the other matrix peptidase and AAA+ ATPase LONP1 that plays a key role for the turnover of respiratory chain components and mtDNA [36, 37, 45, 46], and TFAM as a crucial factor for mtDNA copy number and mtRNA production [47, 48]. Homozygous deletion of *Lonp1* and *Tfam* triggers early embryonal lethality, therefore heterozygous mutants were assessed.

**Fig. 4:**
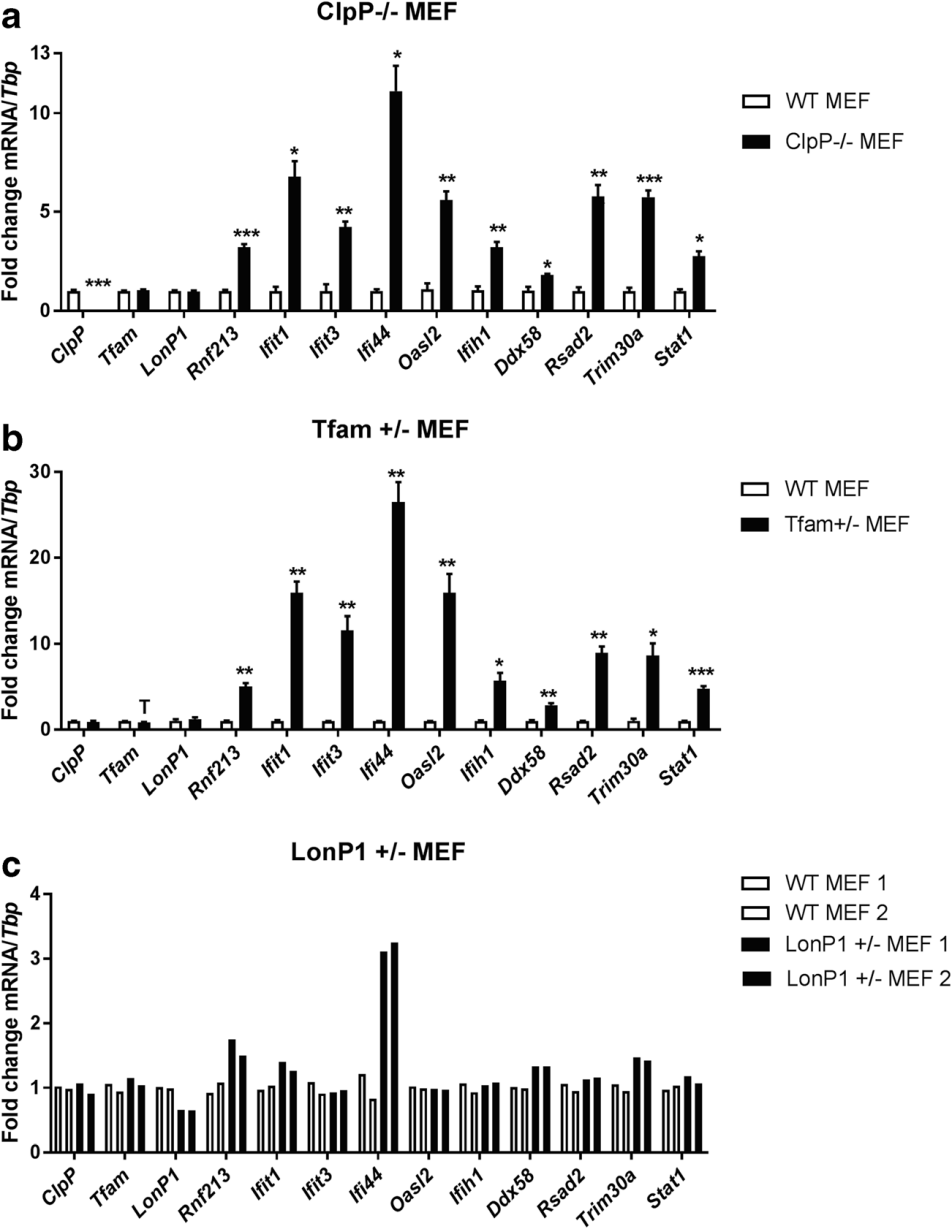
RT-qPCR results for innate immunity related factors in **a***ClpP*^−/−^ MEF (*n* = 4), **b***Tfam*^+/−^ MEF (*n* = 2–4) and **c***Lonp1*^+/−^ MEF (*n* = 2) relative to murine *Tbp* transcript (Trend T 0.05 < *p* < 0.1; *p < 0.05; **p < 0.01; ***p < 0.001)

Quantitative RT-qPCR analysis of mutant MEF and WT controls showed the basal expression of *Rnf213* in *ClpP*^−/−^ cells to be strongly induced as shown before (3.20-fold, *p* = 0.0003) (Fig. 4a), but also *Lonp1*^+/−^ and *Tfam*^+/−^ mutations triggered an upregulation of *Rnf213* (for *Tfam* 5.03-fold, *p* = 0.0017; for *Lonp1* 1.63-fold, *p* = 0.0683 (Fig. 4b, c). *Rnf213* induction was documented in parallel with the transcriptional regulation of sensors for toxic RNA/DNA in the cytosol (*Ifit1*, *Ifit3*, *Oasl2*), of an anti-inflammatory ubiquitin E3 ligase (*Trim30a*, homolog of human RNF88/TRIM5), of several inflammatory factors that relocalize to mitochondria upon innate immunity activation (*Rsad2*, *Ddx58*, *Ifih1, Ifi44*) and their downstream nuclear signal transducer (*Stat1*). Overall, the activation of several innate immunity pathway components was observed also for these two mitochondrial mutants, but it was considerably stronger in *Tfam*^+/−^ than in *Lonp1*^+/−^ cells (Fig. 4).

Upon comparison of FCCP versus Poly(I:C) or LPS effects, and comparing also *Lonp1*^+/−^ versus *Tfam*^+/−^ and *ClpP*^−/−^ effects upon the expression of *Rnf213* and other inflammatory factors, it appears that respiratory failure is less potent for the induction of *Rnf213* than the dysregulation of mitochondrial nucleotides as an immunological trigger.

## Discussion

We had previously observed that the genetic ablation of the mitochondrial peptidase *ClpP* triggers not only accumulation of the AAA+ ATPase ClpX protein in the mitochondrial matrix, but also a transcriptional induction of the AAA+ ATPase *Rnf213* in the cytosol, together with expression upregulations for other innate immunity factors in several mouse organs [12]. The data now reported confirm that also the RNF213 protein abundance is upregulated > 4-fold in *ClpP*^−/−^ brain, while *Lonp1* as the other AAA+ ATPase/peptidase in the mitochondrial matrix and *Tfam* as the main mitochondrial transcription factor show unchanged levels (Fig. 1), indicating a quite selective effect of *ClpP* for *Rnf213*. This *ClpP*-genotype-dependent impact on *Rnf213* now was documented also for immune cells, namely BMDM upon challenge with LPS rather than Pam3CSK4, both of them being simulators of bacterial infection (Fig. S1). It is noteworthy that *ClpP* expression was significantly downregulated upon LPS exposure (Fig. S1). These data provide evidence that ClpP-deficiency triggered problems of mitochondrial protein degradation and folding (mtUPR), due to mutations or infection, are accompanied by a selective nuclear response that provides surplus AAA+ disaggregase capacity to the cytosol.

Trying to understand the mechanism of how a mitochondrial dysfunction might cause this selective cytosolic response, the potency of several stressors on *Rnf213* expression was explored. Neuronal cells were especially sensitive to IFNG, a cytokine that is released from neighboring cells upon immune activation. Endothelial cells appeared particularly responsive to Poly(I:C) exposure. MEF exhibited the strongest response among different cell types to Poly(I:C) and to LPS, as well as the second strongest to IFNG (Figs. 2 and S2). Furthermore, MEF upregulated *Rnf213* upon LPS exposure more than BMDM (Figs. 2 and S1). Overall, only Poly(I:C) triggered a significant induction of *Rnf213* in all cell types, possibly because the RNA sensing pathway is active in every cell type and Poly(I:C) most strongly activates the IRF3 and STAT1/2 transcription factors. IFNG and LPS also activate this response, but the IFNG receptor and TLR4 are more restricted across cell types (Figs. 2 and S2). Thus, murine embryonal fibroblasts at least as much as human umbilical vein endothelial cells may constitute particularly useful tools in the study of neurovascular pathology. This is particularly true for MMD research on intracerebral arteries, whose walls contain endothelial cells in the intima layer and fibroblast cells in the adventitia layer. Given that even the R4859K-RNF213 knock-in mouse model failed to exhibit spontaneous cerebral ischemia or hemorrhage under normal conditions [49, 50], there have to be additional events triggering the MMD stroke phenotypes. Viral infections seem to be plausible triggers in this context.

Poly(I:C) might also be the optimal stressor of the *Rnf213* dependence on mitochondrial ClpP deficiency since Poly(I:C) mimics the activation of TLR3 signaling by toxic dsRNA and since it was recently demonstrated that dysfunctional mitochondria release toxic dsRNA into the cytosol [16]. Diverse mitochondrial mutations and even mild cell stress can contribute to the release of toxic nucleotides from dysfunctional mitochondria via the outer mitochondrial membrane VDAC pore into the cytosol [16, 51]. These new insights are in good agreement with our observations that not only *ClpP* null mutations but also the heterozygous deficiency of the mitochondrial transcription factor *Tfam* and the mitochondrial AAA+ ATPase *Lonp1* upregulate the expression of *Rnf213*, together with several cytosolic RNA/DNA sensors such as *Ifit1*, *Ifit3*, and *Oasl2* (Fig. 4). Among the strongest responses is the ubiquitin E3 ligase *Trim30a* (tripartite motif containing 30α), which is a negative-feedback regulator of the intracellular DNA and DNA virus-triggered response [52]. Other upregulated factors relocalize to mitochondria upon the sensing of toxic nucleic acids (*Rsad2*, *Ddx58*, *Ifih1, Ifi44).* Ifi44 was described as a TLR3-dependent defense factor against RNA virus such as HCV or HIV-1 [53–56], and it was repeatedly found dysregulated in systemic lupus erythematosus (SLE), an autoimmune vasculopathy that is linked to aberrant sensing of self RNA and DNA [57–59]. Interestingly, its homolog *Ifi44l* is associated with an autoimmune vasculopathy named Aicardi-Goutières syndrome (see GeneCards database and [60, 61]). Jointly, the data indicate that the interaction between *ClpP* and *Rnf213* is not specific, but confirm that mutations in several mitochondrial factors activate *Rnf213* among many other interferon stimulated genes. It will be interesting to investigate in future experiments how the ablation of *Rnf213* distorts the innate immune responses.

As a joint downstream factor both for Poly(I:C)-triggered TLR3 signaling and for LPS-triggered TLR4-signaling, the PKR phosphorylation cascade was inhibited by the drug C16 in our experiments. However, only in neuronal cells this approach showed a relevant blockage of *Rnf213* induction and TLR3 dependent *Rsad2* [62], while it failed in MEF and HUVEC (Fig. 3). These data suggest tissue specificity or that further innate immunity pathways are involved. Interestingly, it was observed recently that dysfunctional mitochondria release also toxic mtDNA into the cytosol, thus promoting an SLE-analogous vasculopathy [51], and indeed excess mtDNA is present in *ClpP*^−/−^ tissue [12]. It is known that mtDNA activates TLR9 signaling and is mimicked by CpG administration to cells, but CpG was found to induce *Rnf213* only weakly in BMDM (Fig. S1). Beyond toxic dsRNA signaling that is mimicked by Poly(I:C), it might be worthwhile to explore toxic dsRNA signaling via RIG-I like receptors and the NLRP3 inflammasome, as well as ssRNA signaling that activates TLR7/8 receptors [63].

In zebrafish, it was shown that *Rnf213* knockdown results in abnormal sprouting and irregularities in the intracranial vessel formation, suggesting a role in vascularization [22]. In contrast to this, mice with knockout of *Rnf213* did not show any obvious cerebrovascular phenotypes, only a diminished reactive vascular hyperplasia with significantly thinner intima and medial layers of vessel walls after common carotid artery ligation [64, 65]. After femoral artery ligation in *Rnf213*^−/−^ mice, changes were seen in blood flow and recovery after chronic hind limb ischemia, with angiogenesis being improved [66]. It was also demonstrated that the dysregulation of Mysterin influences the cerebral blood flow after cerebral hypoperfusion [67]. Thus, an attempt to provoke MMD-like phenotypes in these mice via the injection of Poly(I:C) might be rewarding.

Since *Rnf213* expression is triggered by nucleotide toxicity at least as potently as by LPS, it is possible that Mysterin dysfunction in MMD triggers innate immune activation via deficient RNA quality control. Indeed, autoimmune activation in the blood of MMD patients was found to involve elevated levels of CD163 and CXCL5, two IFNG-responsive factors [68–70]. In the latest search of MMD susceptibility genes by genome-wide association analysis, the tissue-enrichment of genes at associated loci were highly expressed in the immune system [71]. One study showed MMD patient circulating endothelial colony-forming cells to exhibit morphological abnormalities of mitochondria with higher radical oxygen species (ROS) as well as elevated Ca^2+^ levels and reduced mitochondrial reductase activity. The authors suggested that MMD might be a mitochondria-related disease [72]. There are other well-known vasculopathies that are caused by impaired nucleotide processing, such as Aicardi-Goutières syndrome that is caused by mutations in the ribonuclease RNASEH2, the exonuclease TREX1, or the deoxynucleoside triphosphate triphosphohydrolase SAMHD1 [73–75], or such as SLE that is triggered by autoimmune responses to toxic DNA or also to mtRNA [76–79]. It is therefore conceivable that they share pathogenetic mechanisms with MMD, which may have to be classified among them.

As a final consideration, in Japan, 80% of MMD patients were reported to carry the RNF213-R1810K mutation, leading to estimates that 2% of the general population there are carriers. However, only 1 out of 150 individuals shows penetrance, so that environmental risk factors such as inflammation were invoked as an explanation [80]. Indeed, infections with the RNA-virus HIV-1 were observed to trigger Moyamoya manifestation [81, 82]. Beyond such ambient trigger factors, our data raise the possibility that subclinical mutations in mitochondrial factors or in the IFNG/TLR3-pathway may contribute to the need of mysterin activation and may enhance the risk for MMD, acting as modifier genes.

## Conclusion

In summary, we demonstrate for the first time that several mutations triggering mitochondrial dysfunction induce mysterin transcriptionally via the innate immune sensing of dsRNA. Mitochondrial dysfunction was previously shown to trigger neuroinflammation, in a chronic process leading to neurodegenerative diseases like Parkinson’s disease (PD) [83–85] or Alzheimer’s disease (AD) [86, 87]. More recently, it became obvious that innate immune reactions are often part of the progression in nervous disorders [88, 89]. Importantly, the deletion of mitochondria/ER-associated innate immunity coordinators like STING or cytosolic immunity sensors like RIPK1 can prevent neurodegenerative processes in PD and the motor neuron disease ALS [90, 91]. The induction of mysterin with its pair of AAA+ ATPase domains and its RING domain may promote the disaggregation and degradation of toxic factors in the cytosol during infectious processes, while its absence may potentiate protein folding problems and the toxicity of associated RNA. Our findings suggest placing *RNF213*-triggered MMD among the vasculopathies that are caused by impaired nucleotide processing, such as Aicardi-Goutières syndrome or SLE.

## Materials and methods

### Mouse breeding

Homozygous *ClpP*^−/−^ and wild-type mice were littermates derived from heterozygous breeder pairs, genotyped, maintained, aged, and dissected as previously described [12]. All animal experiments were performed in compliance with the German animal welfare law and with approval of the local animal authorities (RP Darmstadt, FK/1073).

### Global transcriptome analysis of ClpP^−/−^ mouse tissues

The genome-wide transcriptome profiling effort of 3 WT versus 3 mutant animals was described before [12].

### Global proteome of ClpP^−/−^ mouse brain tissues

Protein abundance of brain tissues was analyzed by label-free quantitative proteomics as recently described [92]. Missing values were replaced by background values from normal distribution.

### In vitro stimulation of bone marrow-derived macrophages

Bone marrow-derived macrophages (BMDM) were generated from bone marrow of 4–5-month-old littermate wild-type and *ClpP*^−/−^ mice. They were cultured on Petri plates in DMEM (Sigma D5796) containing 10% FBS (VWR, 97068-085) plus 30% L929 culture medium for 7 days. BMDM (6 × 10^5^) were seeded in 12-well cell culture plates in DMEM with 10% FBS and 2% conditioned L929, then challenged with LPS (1 μg/ml, tlrl-pb5lps InvivoGen), Pam3CSK (1 μg/ml, tlrl-pms InvivoGen), and CpG (500 nM, ODN1826 tlrl-1826 InvivoGen). RNA was harvested 6 h post-challenge and isolated with Quick-RNA MicroPrep Kit (Zymo Research 11-328 M). Approximately 500 ng RNA was normalized across samples and cDNA was generated using the qScript® cDNA Synthesis Kit (95047-02, Quanta). cDNA was then subjected to qPCR using PerfeCTa SYBR® Green SuperMix (95054, Quanta) and the following primers were used: m*Gapdh*-F (GACTTCAACAGCAATCCCAC) m*Gapdh*-R (TCCACCACCCTGTTGCTGTA), m*Clpp*700-F(TCCAGGCTGGCCTTGAACTC), m*Clpp*920-R(GAGGCCCTGGGAACCAGGAA), m*Rnf213*-F(TTTGTACCGTTCCCCCAAT), m*Rnf213*-R(GTTCACTGCCTCCAATTGCT), m*Rsad2*-F(ATAGTGAGCAATGGCAGCCT), m*Rsad2*-R (AACCTGCTCATCGAAGCTGT). qPCR was run in 384-well plate in CFX384 Real-Time System (BioRad). Three technical replicates were performed for each biological sample, and expression values of each replicate were normalized against *Gapdh* cDNA using the 2^−ΔΔCt^ method [93].

### Derivation and culture of MEF

As reported before [12], each homozygous *ClpP*^−/−^ and WT MEF line was derived from individual littermate embryos at 14.5 days post-coitus of heterozygous breeder pairs. Cells were maintained in Dulbecco’s minimal essential medium 4.5 g/l glucose (Invitrogen) plus 15% fetal bovine growth serum (Gibco, One Shot), 1% Penicillin/Streptomycin (Gibco), 1% Glutamine (Invitrogen) at 37 °C and 5% CO_2_ in a humidified incubator, passaging every 3–4 days. All cell lines were regularly tested for mycoplasma contamination.

### Culture of SH-SH5Y cells

SH-SH5Y cells were cultured in Dulbecco’s minimal essential medium 4.5 g/l glucose (Invitrogen) supplemented with 10% fetal bovine growth serum (Gibco, One Shot), 1% glutamine (Gibco), and 1% penicillin/streptomycin (Gibco), at 37 °C and 5% CO_2_ in a humidified incubator.

### Culture of HUVEC cells

HUVEC cells were grown in Endothelial Cell Growth Medium (Promocell) in tissue flasks, coated with 0.2% gelatin (Sigma) at 37 °C and 5% CO_2_ in a humidified incubator and were passaged every 2–3 days.

### Treatment with FCCP

The uncoupling agent FCCP (trifluoromethoxy carbonylcyanide phenylhydrazone) (Abcam), was administered at 10 μM concentration to MEF (*n* = 4) over 24 h and to human SH-SH5Y neuroblastoma cells (*n* = 5) for 24 h and 36 h and to HUVEC (*n* = 3) for 24 h. Cell pellets were collected and stored at − 80 °C until subsequent RNA extraction.

### Starvation time course of SH-SH5Y cells, HUVEC and MEF

Cells were grown in 6-well plates and medium was switched to HBSS medium (Gibco) without FCS, to subject them to starvation conditions and cells were collected at indicated time points and stored for RNA extraction. Results are shown for 3 different cell lines, processed at the same time.

### Treatment with Poly(I:C)

MEF, SH-SY5Y cells, and HUVEC (*n* = 3–6 each) were cultured as described above and the synthetic dsRNA polymer Poly(I:C) (InvivoGen, HMW/LyoVec) was added to the medium at a concentration of 1 μg/ml for 16 h. Cells were collected for RNA and protein isolation.

### Treatment with LPS

The bacterial cell wall component LPS (InvivoGen, LPS-EB) was administered to the culture medium of MEF, HUVEC, and SH-SY5Y cells (*n* = 3–6,) at 0.1 μg/ml for 24 h. Cells were collected for RNA extraction.

### Treatment with IFNG

Recombinant mouse IFNG (Merck Millipore, IF005) or human IFNG (Preprotech, 300-02) was applied to MEF, SH-SY5Y and HUVEC cells (*n* = 3–6, each) at 50 ng/ml for 24 h. Cells were collected for RNA extraction.

### Treatment with Poly(I:C) and PKR inhibitor C16

SH-SH5Y cells were treated with Poly(I:C) (Invivogen, 1 μg/ml) and C16 (Abcam, 0.5 μM) for 10 h, HUVEC were treated for 16 h with the same concentrations. MEF were treated with Poly(I:C) (1 μg/ml) and C16 (0.5 μM) for 6 h. Cells were collected and RNA was extracted as described above.

### Quantitative real-time RT-qPCR

As in previous analyses [12], TRI reagent (Sigma) was used for isolation of total RNA, and SuperScript IV VILO Master Mix (Invitrogen) for reverse transcription, following manufacturer’s instructions. RT-qPCR was performed with TaqMan Gene Expression Assays (Applied Biosystems) in cDNA from 20 ng total RNA in 20 μl reactions with 2× master mix from Roche in a StepOnePlus Real-Time PCR System (Applied Biosystems). The analysis of the data was carried out with the 2^−ΔΔCT^ method [93]. An RT-qPCR assay of *ClpP* normalized to *Tbp* was used to assess the null mutation in animal tissues and MEF. To quantify mRNA levels, the following TaqMan assays (Applied Biosystems) were employed: *ClpP*-Mm00489940_m1, *Ddx58*-Mm01216853_m1, *DDX58*-Hs01061436_m1, *Eif2ak2*-Mm01235643_m1, *EIF2AK2*-Hs00169345_m1, *HPRT1*-Hs99999909_m1, *Ifi44*-Mm00505670_m1, *Ifih1*-Mm00459183_m1, *IFIH1*-Hs00223420_m1, *Ifit1*-Mm00515153_m1, Ifit3*-*Mm01704846_s1, *Lonp1*-Mm_01236887_m1, *Oasl2*-Mm00496187_m1, *Rnf213*-Mm01248876_m1, *RNF213*-Hs01554655_m1, *Rsad2*-Mm00491265_m1, *RSAD2*-Hs00369813_m1, *Stat1*-Mm00439531_m1, *Tbp*-Mm00446973_m1, *TBP*-Hs99999910_m1, *Tfam*-Mm00447485_m1, *Trim30a*-Mm00493346_m1. Murine TaqMan gene expression assays are indicated with lowercase letters, human TaqMan assays with uppercase.

### Quantitative immunoblotting

Sample preparation for quantitative immunoblotting was done as described before [88]. Samples of 20 μg of protein in 2× Laemmli buffer were heated at 90 °C for 3 min and then separated in 6% tris–glycine polyacrylamide gels, using Precision Plus Protein™ All Blue Standards as size marker. Transfer to nitrocellulose membranes (Protran, GE Healthcare) was done at 20 V over night at 4 °C, with blocking in 5% BSA solution in 1× TBS-T for 1 h at room temperature (RT). Primary antibody incubation against RNF213 (Millipore, ABC1391, 1:1000) and HSP90 (Santa Cruz, sc-7947, 1:1000) occurred in 1× TBS-T solutions overnight at 4 °C. Fluorescence-labeled α-rabbit antibodies (1:15.000, IRDye 680RD, Li-Cor) were used as secondary antibodies. Fluorescence detection occurred with the Li-Cor Odyssey Classic Instrument and bands were densiometrically analyzed with Image Studio Lite, Version 5.2*.* (*n* = 3–4).

### pSILAC time course over 72 h with TMT detection

Mouse embryonic fibroblasts were cultured as described above. Either heavy or light lysine and arginine were added to a final concentration of 73 μg/mL and 42 μg/mL, respectively. For pulse labeling, cells were washed twice with PBS and shifted to heavy medium for the indicated time. Cells were lysed on plate with hot lysis buffer [2% SDS, 50 mM Tris pH 8, 150 mM NaCl, 10 mM TCEP, 40 mM chloracetamide, protease inhibitor tablet (Roche)].

#### Sample preparation

Lysates were sonicated and subjected to methanol-chloroform precipitation. Proteins were resuspended in 8 M Urea, 10 mM EPPS pH 8.2 and diluted to 2 M urea/10 mM EPPS and incubated with 1:50 (wt/wt) LysC (Wako Chemicals) overnight at 37 °C. Digests were further diluted to 1 M Urea and incubated with 1:100 (wt/wt) Trypsin (Promega) for additional 6 h. Peptides were isolated using C18 SepPak columns (Waters) and eluted using 70% acetonitrile. Dried peptides were resuspended in 0.2 M EPPS pH 8.2, 10% acetonitrile for TMT labeling. Fifty micrograms of peptides were incubated with 5 μL TMT reagent for 1 h at room temperature. Reactions were quenched by adding hydroxylamine to a final concentration of 0.5%. Samples were subjected either to high pH fractionation or C18 purified for LC-MS.

#### High-pH reverse phase fractionation

Peptides were either fractionated using a Dionex Ultimate 3000 analytical HPLC or a High pH Reversed phase fractionation kit (ThermoFisher Scientific) according to manufacturer’s instructions. For high pH reversed phase fractionation on the Dionex HPLC, 500 μg of pooled and purified TMT labeled samples were resuspended in 10 mM ammonium-bicarbonate (ABC), 5% ACN, and separated on a 250 mm long C18 column (Aeris Peptide XB-C18, 4.6 mm ID, 2.6 μm particle size; Phenomenex) using a multistep gradient from 100% Solvent A (5% ACN, 10 mM ABC in water) to 60% Solvent B (90% ACN, 10 mM ABC in water) over 70 min. Eluting peptides were collected every 45 s into a total of 96 fractions, which were cross-concatenated into 24 fractions and dried for further processing.

#### Mass spectrometry

Unless stated otherwise, peptides were resuspended in 0.1% FA and separated on an easy nLC 1200 (ThermoFisher Scientific) and a 22 cm long, 75 μm ID fused-silica column, which has been packed in house with 1.9 μm C18 particles (ReproSil-Pur, Dr. Maisch), and kept at 45 °C using an integrated column oven (Sonation). Peptides were eluted by a non-linear gradient from 5 to 38% acetonitrile over 120 min and directly sprayed into a QExactive HF mass-spectrometer equipped with a nanoFlex ion source (ThermoFisher Scientific) at a spray voltage of 2.3 kV. Full-scan MS spectra (350–1400 m/z) were acquired at a resolution of 120,000 at m/z 200, a maximum injection time of 100 ms and an AGC target value of 3 × 10^6^. Up to 20 most intense peptides per full scan were isolated using a 1 Th window and fragmented using higher energy collisional dissociation (normalized collision energy of 35). MS/MS spectra were acquired with a resolution of 45,000 at m/z 200, a maximum injection time of 80 ms and an AGC target value of 1 × 10^5^. Ions with charge states of 1 and > 6 as well as ions with unassigned charge states were not considered for fragmentation. Dynamic exclusion was set to 20 s to minimize repeated sequencing of already acquired precursors.

#### Processing of RAW files—Mass spectrometer

RAW files were analyzed using Proteome Discoverer 2.2 software (ThermoFisher Scientific). Files were recalibrated using the *Mus musculus* SwissProt database (TaxID:10090, version 2017-12-13) with methionine oxidation (+15.995) as dynamic modification and carbamidomethyl (Cys, + 57.021464), TMT6 (N-terminal, + 229.1629) ,and TMT6 (+ 229.1629) at lysines as fixed modifications. Spectra were selected using default settings and database searches performed using SequestHT node in PD. Database searches were performed against trypsin digested *Mus musculus* SwissProt database and FASTA files of common contaminants as quality control. Fixed modifications were set as TMT6 at the N-terminus and carbamidomethyl at cysteine residues. As dynamic modifications TMT6, TMT6+K8 (+ 237.177), Arg10 (+ 10.008), and methionine oxidation were set. After search, error probabilities were calculated using Perlocator with default settings. Consensus Workflow for reporter ion quantification was performed with default settings, except the minimal signal-to-noise ratio was set to 5. Results were then exported to Excel files for further processing.

#### Pulsed SILAC—Half-life evaluation

A custom python script was developed in-house to calculate the half-lives of proteins using an exponential decay curve fit equation: *y* = *y*_0_ + *Ae*^−*x*/*t*^ where *y*_0_is the offset, *A* is the amplitude, and *t* is the time constant. The light peptides were used for the degradation analysis. The *R*^2^ value was used as a quality control measure since *n* = 1 for both WT and knockout time course analysis, and hence, no statistical quantitation could be performed. Peptide filter was set to a *R*^2^ > 0.5. The half-life curve was made to fit all qualifying peptides of a protein providing a robust system with each peptide acting as a replicate and ultimately resulting in a lower but more stringent and robust *R*^2^ value for quality control.

### Statistical evaluation

All expression data were processed with GraphPad software (Version 7.02) and illustrated in bar graphs, showing variance as standard error of the mean (SEM) and *p* values from unpaired Student’s *t* test with Welch’s correction, 1-way ANOVA or 2-way ANOVA. A p value of <0.05 was considered statistically significant.

## Electronic supplementary material

Supplementary Figure S1 In vitro stimulation of murine Bone Marrow-derived Macrophages (BMDM) with the TLR4 agonist LPS, the TLR1/TLR2 agonist Pam3CSK4 and the TLR9 agonist CpG over 6 h demonstrates prominent induction of *Rnf213* together with *Rsad2* and *Ddx58* by LPS, which was further enhanced by the absence of CLPP. Interestingly, the *ClpP* transcript levels were reduced to 31% by LPS exposure. Graphs show statistical results of 2-way ANOVA. * or # *p*<0.05; ** or ## p<0.01; *** or ### *p*<0.001, **** or #### *p*<0.0001. Asterisks show significance compared to untreated WT, hashtags represent significance compared to untreated *ClpP*^-/-^ samples. (PNG 148 kb)

High Resolution (TIF 3177 kb)

Supplementary Figure S2 RT-qPCR results for *Rnf213* transcript in **a)** MEF, **b)** SH-SY5Y and **c)** HUVEC cells after application of the mitochondrial uncoupling agent FCCP. Data are relative to murine *Tbp* or human *HPRT1* transcript levels. (*p<0.05). (PNG 83 kb)

High Resolution (TIF 2485 kb)

Supplementary Figure S3 RNF213 abundance in the presence and absence of ClpP. Graph shows the protein abundance of RNF213 determined by mass-spectrometry over a time-course of 72 h in WT (blue) and ClpP^-/^^-^ (red) MEF (*n*=1). Each point represents a signal for a different peptide. (PNG 115 kb)

High Resolution (TIF 220 kb)

Supplementary Table S1 Different sheets summarize fold-changes and *p*-values for all expression analyses displayed in the diverse figures. (XLSX 21 kb)
