# Supplementary figures and images for: Loss of mitochondrial ClpP, Lonp1, and Tfam triggers transcriptional induction of Rnf213, a susceptibility factor for moyamoya disease
Source: Neurogenetics. 2020 Apr 28;21(3):187–203. doi: 10.1007/s10048-020-00609-2 (PMC7283203; doi:10.1007/s10048-020-00609-2)

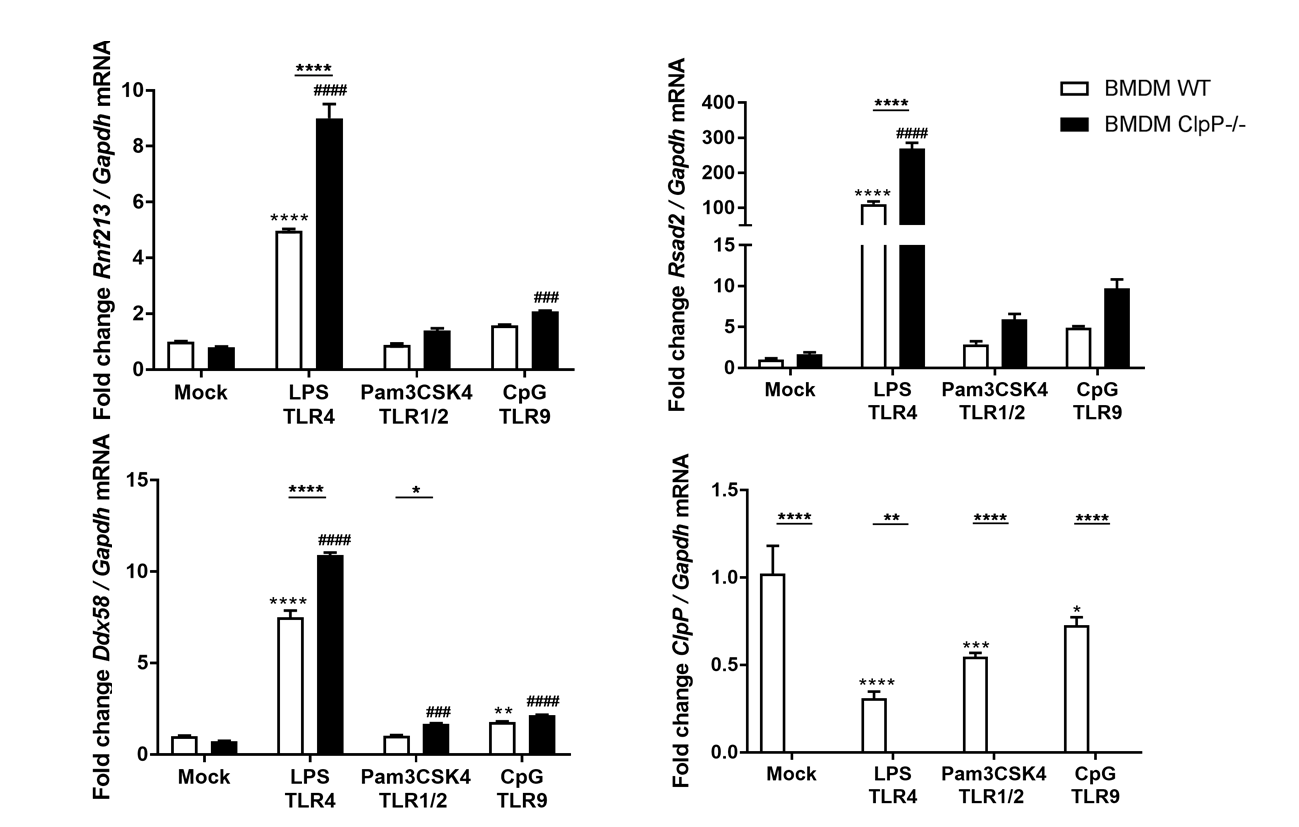

Supplement: Supplementary file 1 — In vitro stimulation of murine Bone Marrow-derived Macrophages (BMDM) with the TLR4 agonist LPS, the TLR1/TLR2 agonist Pam3CSK4 and the TLR9 agonist CpG over 6 h demonstrates prominent induction of Rnf213 together with Rsad2 and Ddx58 by LPS, which was further enhanced by the absence of CLPP. Interestingly, the ClpP transcript levels were reduced to 31% by LPS exposure. Graphs show statistical results of 2-way ANOVA. * or # p<0.05; ** or ## p<0.01; *** or ### p<0.001, **** or #### p<0.0001. Asterisks show significance compared to untreated WT, hashtags represent significance compared to untreated ClpP-/- samples. (PNG 148 kb) [file 10048_2020_609_Fig4_ESM.png]

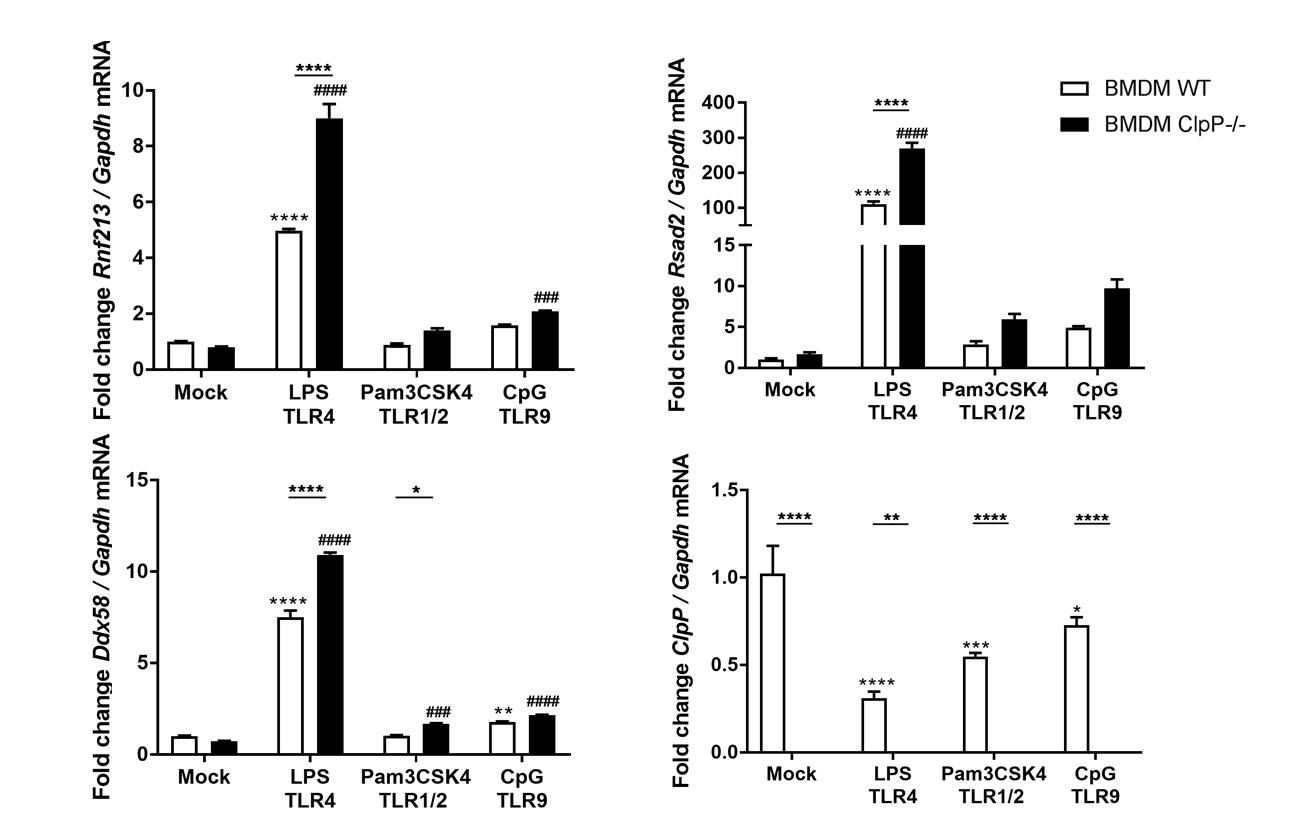

Supplement: Supplementary file 2 — High Resolution (TIF 3177 kb) [file 10048_2020_609_MOESM1_ESM.tif]

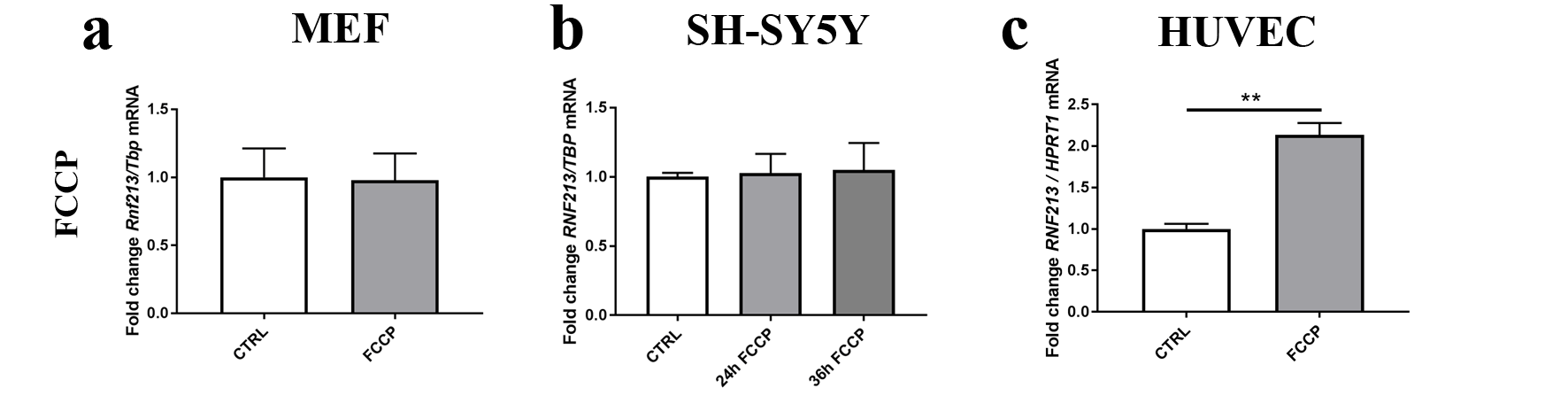

Supplement: Supplementary file 3 — RT-qPCR results for Rnf213 transcript in a) MEF, b) SH-SY5Y and c) HUVEC cells after application of the mitochondrial uncoupling agent FCCP. Data are relative to murine Tbp or human HPRT1 transcript levels. (*p<0.05). (PNG 83 kb) [file 10048_2020_609_Fig5_ESM.png]

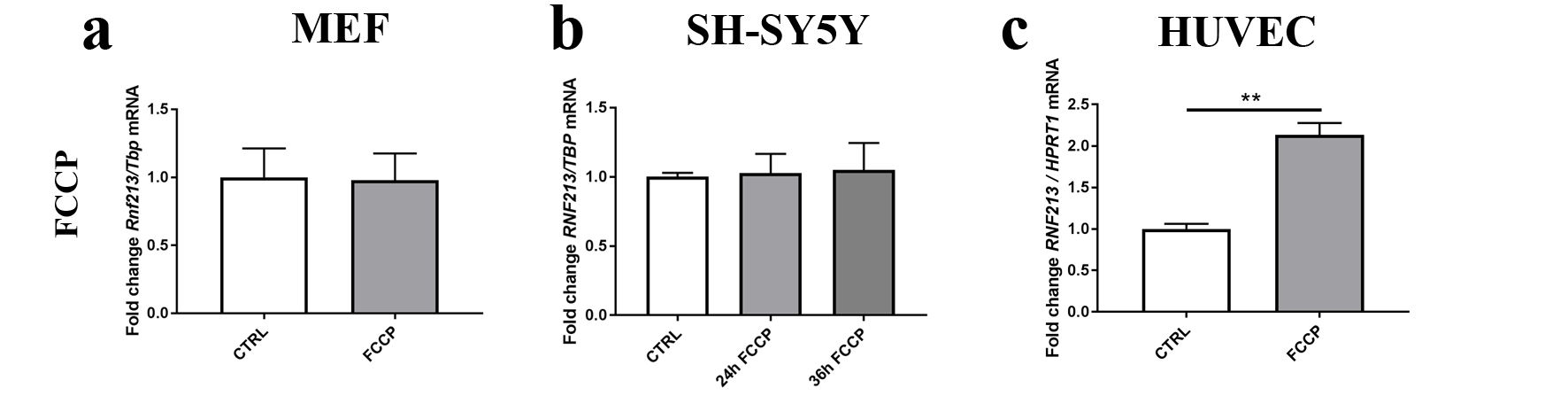

Supplement: Supplementary file 4 — High Resolution (TIF 2485 kb) [file 10048_2020_609_MOESM2_ESM.tif]

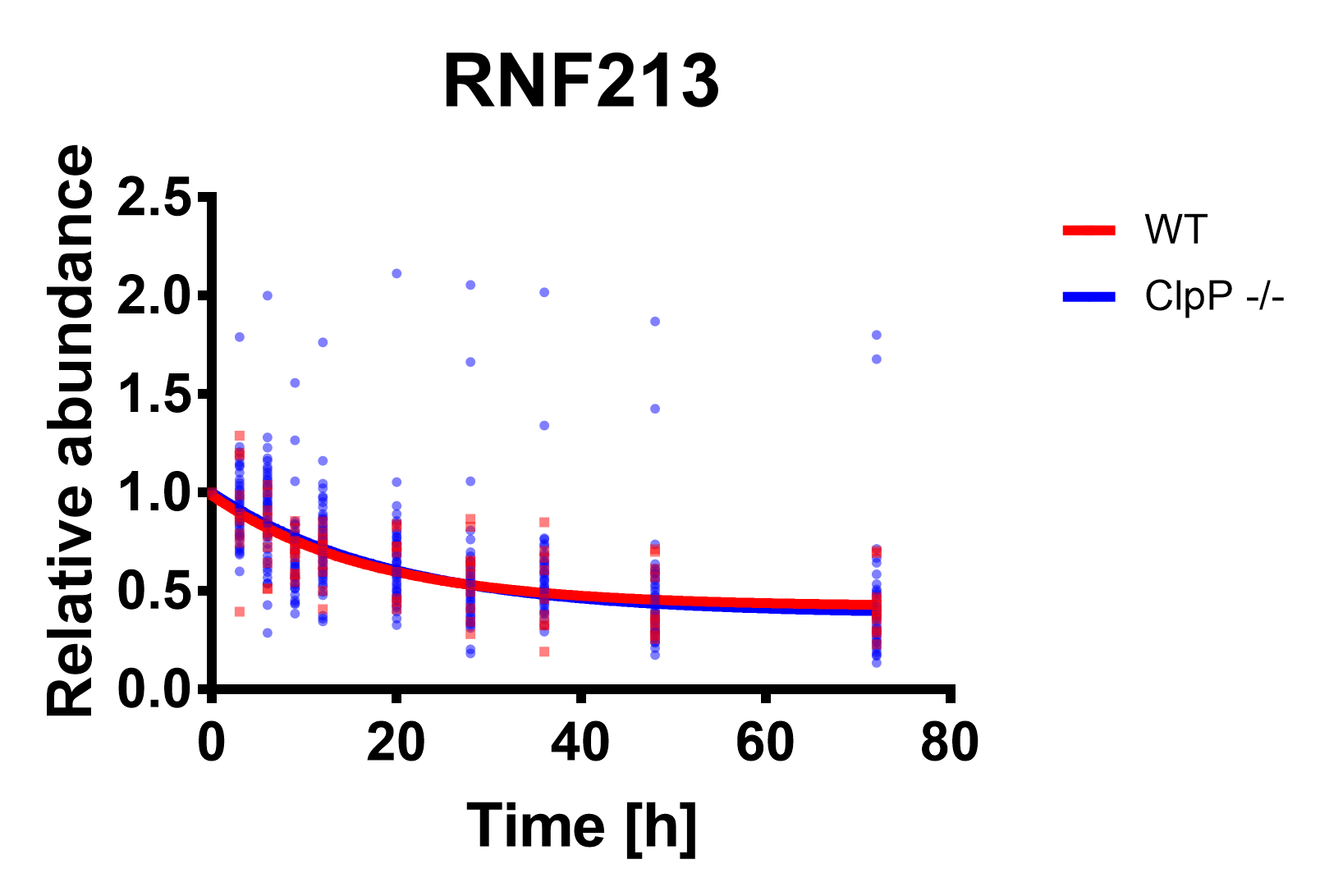

Supplement: Supplementary file 5 — RNF213 abundance in the presence and absence of ClpP. Graph shows the protein abundance of RNF213 determined by mass-spectrometry over a time-course of 72 h in WT (blue) and ClpP-/- (red) MEF (n=1). Each point represents a signal for a different peptide. (PNG 115 kb) [file 10048_2020_609_Fig6_ESM.png]

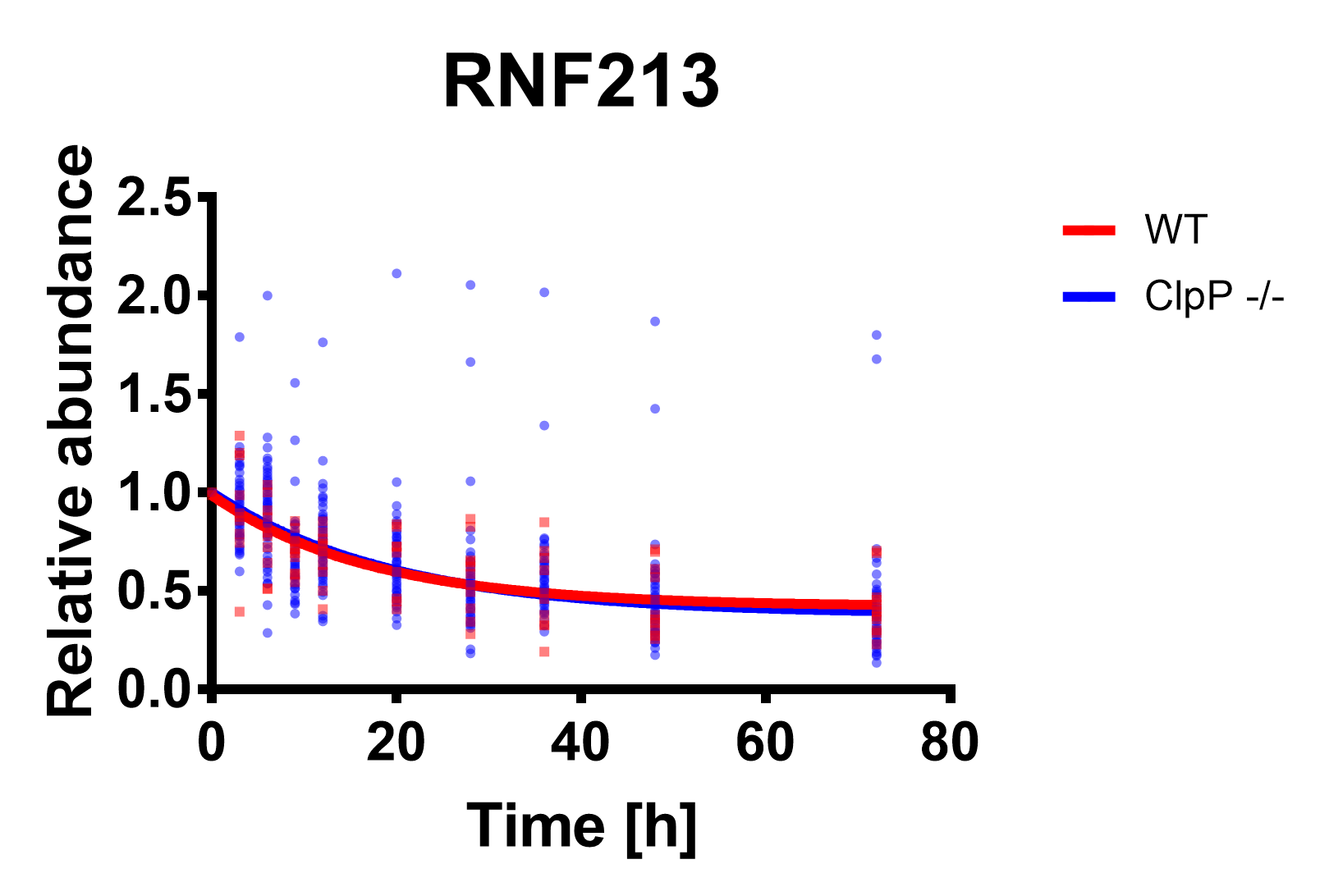

Supplement: Supplementary file 6 — High Resolution (TIF 220 kb) [file 10048_2020_609_MOESM3_ESM.tif]
